# The pharmacokinetics/pharmacodynamics index of high-dose tigecycline in patients with carbapenem-resistant Enterobacterales bloodstream infection: a prospective study

**DOI:** 10.1186/s12879-026-13601-2

**Published:** 2026-05-20

**Authors:** Sirapat Somsirikarnjanakoon, Worapong Nasomsong, Jatapat Hemapanpairoa, Piraporn Juntanawiwat, Wichai Santimaleeworagun

**Affiliations:** 1 The College of Pharmacotherapy of Thailand, Nonthaburi, 11000 Thailand; 2 Division of Clinical Pharmacy, Faculty of Pharmaceutical Sciences, Burapha University, Chonburi, 20131 Thailand; 3 Division of Infectious Disease, Department of Medicine, Phramongkutklao Hospital and Phramongkutklao College of Medicine, Bangkok, 10400 Thailand; 4 Division of Pharmaceutical care, Faculty of Pharmacy, Silpakorn University, Nakorn Pathom, 73000 Thailand; 5 Pharmaceutical Inititiative for Resistant Bacteria and Infectious Disease Working Group (PIRBIG), Nakorn Pathom, 73000 Thailand; 6 Department of Clinical Pathology, Division of Microbiology, Phramongkutklao Hospital, Bangkok, 10400 Thailand

**Keywords:** Tigecycline, High-dose tigecycline, *Klebsiella pneumoniae*, Bloodstream infections, Pharmacokinetics/Pharmacodynamics

## Abstract

**Background:**

Carbapenem-resistant Enterobacterales (CRE) bloodstream infections (BSIs) are a critical public health threat with high mortality rates. In Thailand, limited access to novel antimicrobial agents restricts first-line therapeutic options, especially for NDM-producing CRE. While tigecycline (TGC) retains in vitro activity against all carbapenemase classes, its large volume of distribution compromises serum concentrations, making its efficacy in bacteremia highly controversial. Therefore, the objective of this study was to identify the optimal pharmacokinetic/pharmacodynamic (PK/PD) targets of high-dose tigecycline (HD-TGC) that are predictive of favorable clinical and microbiological outcomes in patients with CRE BSIs.

**Methods:**

We conducted a prospective study at a tertiary-care teaching hospital between May 2023 and May 2025, enrolling patients receiving HD-TGC for the treatment of CRE BSIs. For the non-compartmental pharmacokinetic analysis, TGC plasma concentrations were collected at five time points from each patient following at least 36 h of HD-TGC administration. The steady-state 24-hour area under the concentration-time curve (AUCss, 0–24 h) was calculated using Phoenix WinNonlin software. In vitro susceptibility was determined by the broth microdilution method. Patients completing a minimum of 48 h of therapy were evaluated for clinical and microbiological outcomes, and the relationships between the steady-state area under the curve to the minimum inhibitory concentration (AUCss, 0–24 h/MIC) and these outcomes were examined.

**Results:**

Clinical outcomes were evaluated in 30 patients; all-cause mortality rates were 16.7% at day 7, 50.0% at day 14, and 80.0% at day 30. Pharmacokinetic analysis of 137 plasma concentrations from 28 patients revealed a median AUCss, 0–24 h of 14.81 mg·h/L. A PK/PD target of AUCss, 0–24 h/MIC **≥** 9.90 was identified as significantly associated with decrease in CRE-attributable mortality and increase in the microbiological eradication rate. The causative CRE isolates demonstrated MIC50 and MIC90 values of 0.5 mg/L and 1 mg/L, respectively.

**Conclusions:**

This study proposes an exploratory, hypothesis-generating AUCss, 0–24 h/MIC target of **≥** 9.90, which is associated with favorable clinical and microbiological outcomes in CRE BSIs treated with HD-TGC. To achieve this target, our data suggest that HD-TGC is most likely to be effective against *Klebsiella pneumoniae* with a TGC MIC ≤ 1 mg/L.

**Clinical trial number:**

Not applicable.

**Supplementary Information:**

The online version contains supplementary material available at 10.1186/s12879-026-13601-2.

## Background

Carbapenem-resistant Enterobacterales (CRE) constitute an urgent global public health problem. World Health Organization (WHO) included CRE as a critical priority group from 2017 to 2024 [1]. Bloodstream infections (BSIs) caused by CRE are associated with a high mortality rate, often reported to range from 40% to 70% [2–4]. In Thailand, data from the National Antimicrobial Resistance Surveillance Center (NARST) indicate a significant rise in the prevalence of Carbapenem-resistant *Klebsiella pneumoniae* (CR-KP), which increased from 1.5% in 2015 to 16.0% in 2023 [5]. Among carbapenemase-producing CRE (CP-CRE) isolates in the country, NDM is the most commonly identified carbapenemase (47.33%), followed by OXA-48 (43.33%), with NDM plus OXA-48 accounting for 6.67% [6]. While recent guidance, including the 2024 Infectious Diseases Society of America (IDSA) [7] and the European Society of Clinical Microbiology and Infectious Diseases (ESCMID) guidelines [8], recommend ceftazidime-avibactam, either alone or in combination with aztreonam, as a first-line treatment for CRE BSIs. This recommendation is based on superior outcomes, including lower 30-day mortality and nephrotoxicity rates, compared to colistin-based regimens [9, 10]. However, ceftazidime-avibactam has limited activity against NDM-producing CRE. Furthermore, access to aztreonam for combination therapy with ceftazidime-avibactam against NDM producing CRE is restricted in Thailand. Consequently, older antimicrobial agents continue to play a crucial role in the treatment of CRE infections, with CR-KP susceptibility reported as 97.9% for amikacin and 79.6% for tigecycline (TGC), while colistin shows an intermediate susceptibility rate of 67.3% [11]. Notably, TGC exhibits in vitro activity against all types of CP-CRE [8].

TGC is a member of the glycylcycline class of antibiotics. It is approved by the United States Food and Drug Administration (U.S. FDA) for treatment of complicated intra-abdominal infection (cIAI), complicated skin and skin structure infection (cSSTI), and community acquired pneumonia (CAP) with standard-dose tigecycline (SD-TGC) comprises an initial loading dose 100 mg followed by 50 mg administered intravenously every 12 h [12]. However, administration of high-dose tigecycline (HD-TGC), consisting of a 200 mg loading dose followed by 100 mg every 12 h, has been shown to improve clinical cure in critically ill patients [13, 14] and reduce mortality in those with CR-KP BSIs [15, 16].

Nevertheless, no previous studies have evaluated the pharmacokinetics/pharmacodynamics (PK/PD) target for the treatment of CRE BSIs using HD-TGC. Therefore, the objective of this study is to investigate the optimal PK/PD target of HD-TGC for the treatment of CRE BSIs, with the ultimate goal of optimizing therapeutic efficacy.

## Methods

### Study design and participants

This prospective, single-center study was conducted at Phramongkutklao Hospital, a tertiary-care teaching hospital in Bangkok, Thailand, between May 2023 and May 2025. The study included patients over 20 years of age who met the following criteria: (1) admitted to the hospital with a CRE BSIs documented by blood culture, (2) treated with HD-TGC, defined as a 200 mg loading dose, followed by a 100 mg maintenance dose via 60-minute IV infusion every 12 h. Patients were required to receive HD-TGC for a minimum of 36 h prior to sampling to enable pharmacokinetic analysis. Patients were excluded from the study if any of the following conditions were present: (1) pregnancy, (2) declined to provide informed consent for plasma sampling, and (3) non-compliance with the dosing regimen. To determine the clinical and microbiological outcomes, only patients who received HD-TGC therapy for at least 48 h were included in the outcome analysis.

### Ethic approval

The study protocol was reviewed and approved by the Institutional Review Board (IRB) of the Royal Thai Army Medical Department at Phramongkutklao College of Medicine and Phramongkutklao Hospital. Blood samples and data collection were conducted only after obtaining this ethical approval and permission from the hospital director.

Written informed consent was obtained from all participants prior to enrollment in the study.

### Blood sampling, TGC assay, and pharmacokinetic analysis

For pharmacokinetic analysis, five 2–3 mL blood samples were collected from patients receiving HD-TGC for at least 36 h. Sampling time points relative to the start of infusion included: 1 h, > 1 to < 4 h, > 4 to < 6 h, > 6 to < 12 h, and the trough (before the next dose). The collected blood was centrifuged at 3,000 rpm for 10 min at 4℃, and the serum was collected and stored at − 80℃ until analysis.

TGC serum concentrations were quantified using a Liquid Chromatography-Tandem Mass Spectrometry (LC-MS/MS) method (Agilent 1100 series LC/MSD Trap Operations), adapted from a previously published method [17]. Serum samples, after being thawed at room temperature, underwent protein precipitation by mixing a 100 µL plasma aliquot with 600 µL of methanol and centrifuging the mixture at 10,000 rpm for 10 min. The supernatant was collected, filtered through a 0.22 μm nylon syringe filter, and a 40 µL volume was subsequently injected. Separation utilized a Poroshell 120 EC-C18 column (3.0 × 150 mm) (Agilent), with a mobile phase consisting of 0.1% formic acid (Fisher Chemical™) and methanol (Merck) via gradient elution at a 0.4 mL/min flow rate. Detection was performed in positive electrospray ionization (ESI) mode using Multiple Reaction Monitoring (MRM), monitoring the m/z 586.3 → 513.2 transition at a retention time of 7.3 min. Quantification was based on an eight-point calibration curve (0.05 to 2.5 mg/L) with a validated linear regression equation (r^2^ = 0.9991), demonstrating a Lower Limit of Quantification (LLOQ) of 0.05 mg/L and a coefficient of variation < 10%.

Pharmacokinetic analysis was performed using Phoenix WinNonlin version 8.5. The evaluation utilized non-compartmental pharmacokinetic analysis to determine the area under the concentration-time curve over the 24-hour dosing interval at steady-state (AUCss, 0–24 h).

### Data collection, and outcomes

Patient data were collected prospectively upon enrollment and throughout the hospitalization period via dedicated case report forms and subsequently entered into a secure research database. Variables were systematically recorded across five domains: (1) demographic and baseline characteristics including age, sex, body weight, height, underlying disease, and the Charlson Comorbidity Index; (2) infection and treatment data documenting the source of infection, the specific CRE species, antimicrobial culture sensitivity results, presence of polymicrobial infection, the specific antibiotic treatment regimen for CRE bacteremia, and achievement of source control; (3) severity of illness was assessed at the time of positive CRE blood culture by presence of septic shock, critically ill status, mechanical ventilation and vasopressor use, and validated SOFA and APACHE II scores; (4) laboratory data including complete blood count, liver and renal function tests, arterial blood gas, and electrolytes; and (5) drug administration and monitoring with precise documentation of HD-TGC dosing regimens and therapeutic drug monitoring data, including the exact date and time of administration and plasma sample collection.

Clinical outcomes were measured as all-cause mortality assessed at 7, 14, and 30 days post-index culture. CRE-attributable mortality at 14 days, 30 days and in-hospital mortality was defined as death directly related to the CRE infection. The criteria required a laboratory-confirmed CRE BSI and the absence of an alternative primary cause of death (e.g., advanced-stage malignancy or severe cardiovascular disease). Causality was independently adjudicated by an infectious disease physician blinded to the plasma TGC level. Clinical failure was defined as a composite endpoint including death or patients who received add-on therapy for CRE BSIs or the presence of clinical signs and symptoms of worsening infection after at least 48 h of HD-TGC therapy. Microbiological failure was defined by a persistent positive blood culture at least 48 h after the initiation of HD-TGC therapy, Patients who died before repeat cultures were excluded.

### Microbiological data

CRE were initially identified from blood culture specimens by the hospital’s laboratory using automated susceptibility testing (Sensititre™ Aris HiQ System). The minimum inhibitory concentration (MIC) of TGC was subsequently determined by the broth microdilution method, which is considered the gold standard. The procedure was adapted from a previously published method [18]. Susceptibility to TGC was classified according to the breakpoints established by both the U.S. FDA (susceptible: MIC ≤ 2 mg/L) [19] and the European Committee on Antimicrobial Susceptibility Testing (EUCAST) (susceptible: MIC ≤ 0.5 mg/L) [20].

### PK/PD

The ratio of the steady-state area under the curve to the minimum inhibitory concentration (AUCss, 0–24 h/MIC) was evaluated as the PK/PD index to predict clinical and microbiological outcomes for HD-TGC. This analysis investigated the correlation between individual AUCss, 0–24 h and the MIC of the causative isolate.

### Statistical analysis

Descriptive statistics were used to summarize baseline patient characteristics and the MIC distribution for the study organism was characterized by determining the 50th percentile (MIC50) and 90th percentile (MIC90). The optimal AUCss, 0–24 h/MIC ratio cutoff value for predicting TGC treatment outcome was identified using Classification and Regression Tree (CART) analysis and Receiver Operating Characteristic (ROC) curves, utilizing Youden’s Index to identify the precise value. Treatment outcomes and the derived AUCss, 0–24 h/MIC cutoff point were compared using the Chi-square test and the Fisher’s exact test, with P-value < 0.05 considered statistically significant. The risk factors were analyzed using univariable logistic regression and the univariable with *p* < 0.05 were included in the multivariable logistic regression models. All analyses were performed using Statistical Package for the Social Sciences (SPSS) version 27.0 (IBM TechXchange Community; Armonk, New York, USA).

## Results

### Baseline characteristics

The patient enrollment and study flow are detailed in Supplementary Fig. 1. The cohort comprised 30 patients, predominantly male (83.33%) with a median age of 73.5 years. Most of the patients were critically ill (96.7%) with 56.7% presenting with septic shock. The primary sources of the CRE BSIs were catheter-related BSIs (40%), followed by intra-abdominal infections (30%) and pneumonia (26.67%). The predominant CRE pathogen was *Klebsiella pneumoniae* (96.67%). The determined MIC50 and MIC90 for TGC were 0.5 mg/L and 1 mg/L, respectively, within an overall range of 0.25 to 2 mg/L. For definitive treatment, the most frequent regimens were colistin-based combinations (46.67%), followed by amikacin-based combinations (26.67%) and imipenem-based combinations (13.33%) (Table 1).

**Table 1:** Baseline characteristics of patients with carbapenem resistant Enterobacterales bloodstream infection (*n* = 30 participants)

Characteristics	Values	
Male, n (%)	25 (83.33)	
Age, median (IQR) (years)	73.5 (60.25-85.00)	
Body weight, median (IQR) (kg)	60 (52.75-70.00)	
Body mass index, median (IQR) (kg/m^2^)	22.59 (19.33–24.86)	
Albumin, median (IQR) (mg/dL)	2.49 (2.03–2.83)	
**Source of bacteremia**, n (%)		
Catheter-related	12 (40.00)	
Intra-abdominal	9 (30.00)	
Pneumonia	8 (26.67)	
Urinary tract	2 (6.67)	
Primary bacteremia	2 (6.67)	
Skin and soft tissue	1 (3.33)	
Bone and joint	1 (3.33)	
**Comorbidities**		
Hypertension, n (%)	20 (66.67)	
Malignancy, n (%)	15 (50.00)	
Diabetes, n (%)	11 (36.67)	
Chronic kidney disease, n (%)	10 (33.33)	
Cardiovascular disease, n (%)	7 (23.33)	
Cerebrovascular disease, n (%)	4 (13.33)	
Cirrhosis, n (%)	3 (10.00)	
Immunosuppressive agents, n (%)	5 (16.67)	
Charlson comorbidity index, median (IQR)	5 (4.00-6.25)	
**Severity score**, median (IQR)		
APACHE II score	26.5 (21.75-31.00)	
SOFA score	8.5 (6.00-11.25)	
**Severities**, n (%)		
Critically ill	29 (96.67)	
Septic shock	17 (56.67)	
Mechanical ventilation	21 (70.00)	
Renal replacement therapy	10 (33.33)	
**Definitive treatment**		
HD-TGC + colistin, n (%)	14 (46.67)	
HD-TGC + amikacin, n (%)	8 (26.67)	
HD-TGC + imipenem, n (%)	4 (13.33)	
HD-TGC + ceftazidime/avibactam, n (%)	3 (10.00)	
HD-TGC + fosfomycin, n (%)	1 (3.33)	
Time to receive ≥ 1 active agent, median (IQR) (hours)	45.96 (2.06–79.47)	
Time to receive HD-TGC combination, median (IQR) (hours)	77.91 (56.02–91.52)	
Duration of HD-TGC, median (IQR) (days)	8 (5.75-10.00)	
Time to first TGC concentration sampling, median (IQR) (hours)	61.21 (45.62–88.63)	
Source control, n (%)	20 (66.67)	
**Pathogens**, n (%)		
CR-KP	29 (96.67)	
CoR-KP	9 (30.00)	
CR-*Enterobacter kobei*	1 (3.33)	
**MIC** (mg/L), n (%)		
0.25	8 (26.67)	
0.5	7 (23.33)	
1.0	12 (40.00)	
2.0	3 (10.00)	
MIC50	0.5	
MIC90	1	

APACHE-II: Acute physiology and chronic health evaluation II, SOFA: Sequential organ failure assessment HD-TGC: high-dose tigecycline, CR-KP: carbapenem-resistant *Klebsiella pneumoniae*, CoR-KP: colistin-resistant *Klebsiella pneumoniae*, CR-*Enterobacter kobei*: carbapenem-resistant *Enterobacter kobei*, MIC: minimum inhibitory concentration

### Pharmacokinetics of HD-TGC

A total of 137 TGC concentrations from 28 patients were used to evaluate non-compartmental pharmacokinetics. Two patients were excluded from the analysis due to death during plasma collection. This prevented the acquisition of the required three time points in the elimination phase, thereby precluding the calculation of steady-state parameters. The pharmacokinetic profiles demonstrated a maximum plasma concentration (Cmax) with a mean ± standard deviation (SD) of 0.83 ± 0.57 mg/L. The trough concentration (Ctr) exhibited a mean ± SD of 0.23 ± 0.16 mg/L. Finally, the AUCss, 0–24 h was calculated to have a mean ± SD of 19.47 ± 16.56 mg·h/L and a median of 14.81 (9.48–23.50) mg·h/L, as detailed in Supplementary Table 1.

### The clinical and microbiological outcomes

Clinical outcomes were assessed for the 30 patients who received HD-TGC therapy for at least 48 h (Table 2). All-cause mortality rates were 16.67% at day 7, 50% at day 14, and 80% at day 30. The CRE-attributable mortality rates at 14 days, 30 days, and in-hospital were 13.33%, 33.33%, and 40%, respectively. The clinical cure rate was 70% at day 7 but decreased to 36.67% by day 14. The microbiological cure rates at day 7 and day 14 were 71.43% and 88.89%, respectively. The clinical and microbiological outcomes stratified by TGC MIC were evaluated and are presented in Supplementary Table 2.

**Table 2:** Clinical and microbiological outcomes

Clinical and microbiological outcomes	Number of patients (percentage)
**Mortality**	
7-day all-cause mortality	5/30 (16.67)
14-day all-cause mortality	15/30 (50.00)
30-day all-cause mortality	24/30 (80.00)
14-day CRE-attributable mortality	4/30 (13.33)
30-day CRE-attributable mortality	10/30 (33.33)
In-hospital CRE-attributable mortality	12/30 (40.00)
**Clinical response**	
7-day clinical cure	21/30 (70.00)
14-day clinical cure	11/30 (36.67)
**Microbiological response**	
7-day microbiological cure	20/28 (71.43)
14-day microbiological cure	16/18 (88.89)

### The PK/PD index and clinical outcomes

#### Derivation of the PK/PD cutoff

**Fig. 1:**
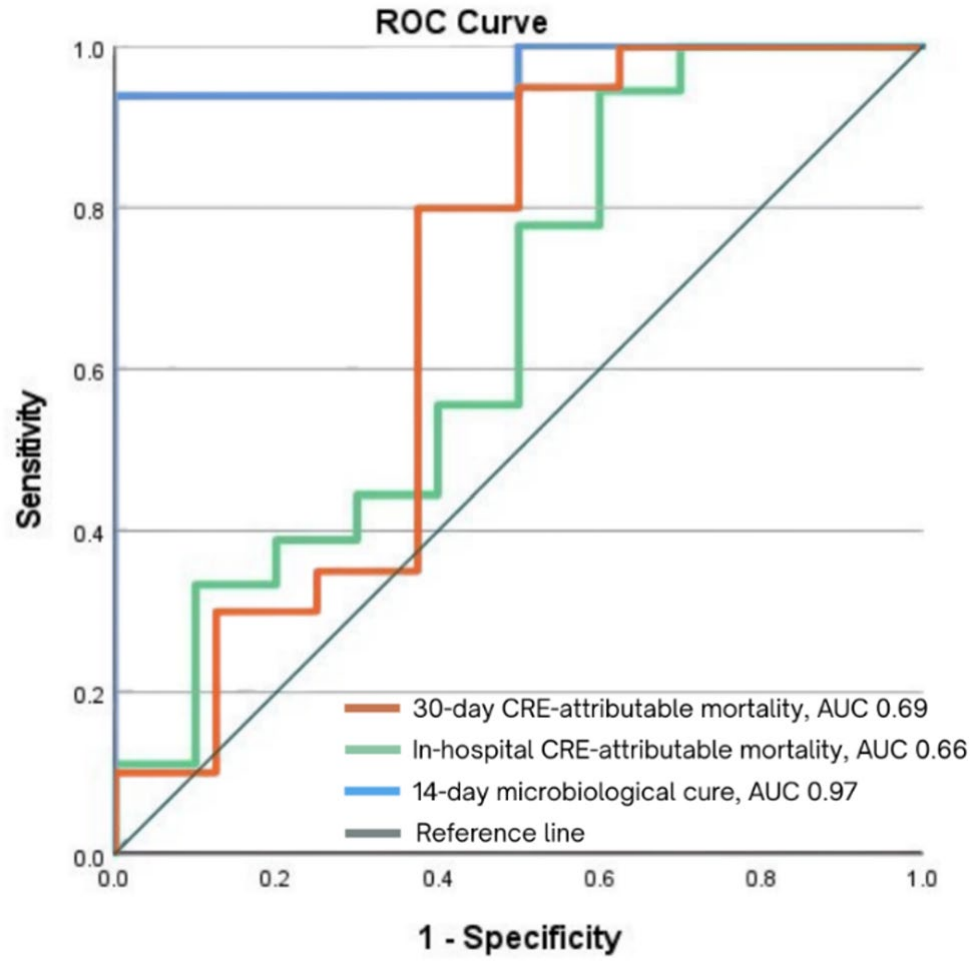
ROC curves of AUCss, 0–24 h/MIC predicting mortality and microbiological outcomes

ROC curve analysis was performed using data from the 28 patients with complete pharmacokinetic profiles to evaluate the AUCss,0–24 h/MIC ratio as a predictor of clinical and microbiological outcomes (Fig. 1). The index demonstrated excellent discriminative performance for 14-day microbiological cure, yielding an AUC of 0.97 (95% CI: 0.884–1.000) with an optimal breakpoint of 9.58. regarding mortality outcomes, the predictive capacity was moderate; the AUC for 30-day CRE-attributable mortality was 0.69 (95% CI: 0.430–0.945) at a cutoff of 9.85, while in-hospital CRE-attributable mortality showed an AUC of 0.66 (95% CI: 0.431–0.880) at a cutoff of 9.85.

#### Performance assessment of the derived cutoff

An AUCss,0–24 h/MIC ratio of 9.90 was determined to be the optimal breakpoint for predicting these clinical outcomes. The predictive performance of this threshold for 14-day microbiological cure yielded a ROC AUC of 0.75 (100% sensitivity, 50% specificity). For 30-day CRE-attributable mortality, the ROC AUC was 0.80 (85.7% sensitivity, 25% specificity), and for in-hospital CRE-attributable mortality, the ROC AUC was 0.73 (71.4% sensitivity, 25% specificity).

Clinical outcomes were explicitly compared based on PK/PD target attainment. Patients who successfully achieved the AUCss, 0–24 h/MIC ≥ 9.90 target (*n* = 23) demonstrated significantly lower mortality rates compared to those who did not achieve this threshold (*n* = 5). Specifically, the target-attainment group exhibited a substantial reduction in both 30-day CRE-attributable mortality (17.4% [4/23] vs. 80.0% [4/5]; *p* < 0.05) and in-hospital CRE-attributable mortality (26.1% [6/23] vs. 80.0% [4/5]; *p* < 0.05). Furthermore, microbiological eradication was achieved in 100% (14/14) of patients who met the target, compared to 50% (2/4) of those who did not (*p* < 0.05). These findings are detailed in Table 3.

In the univariable analysis, variables significantly associated with 30-day CRE-attributable mortality included the APACHE II score (*p* = 0.03) and an AUCss, 0–24 h/MIC ≥ 9.90 (*p* = 0.02). Subsequent multivariable analysis demonstrated that achieving an AUCss, 0–24 h/MIC ≥ 9.90 (OR 0.04, 95% CI 0.002–0.780, *p* = 0.03) was an independent protective factor against 30-day CRE-attributable mortality (Supplementary Table 3).

**Table 3:** Comparison of clinical and microbiological outcomes based on the AUCss, 0–24 h/MIC breakpoint of 9.90

Clinical and microbiological outcomes	AUC_ss, 0–24 h_/MIC ≥ 9.90	AUC_ss, 0–24 h_/MIC < 9.90	*P*-value	OR (95%CI)
14-day CRE-attributable mortality	3/23 (13%)	1/5 (20.0%)	0.568	0.60 (0.049–7.345)
30-day CRE-attributable mortality	4/23 (17.4%)	4/5 (80.0%)	0.015*	0.053 (0.005–0.605)
In-hospital CRE-attributable mortality	6/23 (26.1%)	4/5 (80.0%)	0.041*	0.088 (0.008–0.954)
14-day clinical cure	9/23 (39.1%)	2/5 (40.0%)	0.671	1.037 (0.144–7.477)
14-day microbiological cure	14/14 (100%)	2/4 (50.0%)	0.039*	N/A

* *p* < 0.05, OR: odd ratio, N/A: not applicable

## Discussion

The pharmacokinetic profile of TGC has limited its clinical utility for BSIs, primarily due to an extensive volume of distribution (Vd) that results in low plasma concentrations. In the present study, the mean and median AUCss, 0–24 h were 19.47 mg·h/L and 14.81 mg·h/L, respectively. Our median value is considerably higher than the 3.61 mg·h/L reported by De Pascale et al. [21]. This discrepancy is likely driven by the higher disease severity in their cohort compared to ours. Their patients exhibited a lower median Cmax (0.34 mg/L) and Ctr (0.09 mg/L), alongside a significantly larger Vd (438.6 L) and clearance (42.1 L/h), all of which contribute to lower overall drug exposure. Conversely, our AUCss, 0–24 h values are lower than other published data from cohorts with lower disease severity, which reported mean AUCss, 0–24 h values of 30.58 mg·h/L and 25.78 mg·h/L [22, 23]. These variations are likely attributable to differences in patient acuity. It is well documented that the severe pathophysiology of critical illness alters TGC disposition by increasing Vd or clearance [24]. Furthermore, another factor contributing to the lower exposures in our cohort is that seven patients underwent pharmacokinetic sampling before reaching steady state. Nevertheless, it should be noted that the extent of drug accumulation may be diminished in patients with high disease severity, particularly those presenting with sepsis or septic shock [25].

The clinical outcomes observed in our cohort were characterized by 7-day, 14-day, and 30-day all-cause mortality rates in our cohort were 16.67%, 50%, and 80%, respectively. Our 30-day mortality rate is notably high compared to a previous study of HD-TGC for CR-KP BSIs, which reported an in-hospital mortality rate of 52.2% [15]. This poor outcome is likely multifactorial and reflects the high severity of our patient cohort, which was characterized by advanced age, a high median APACHE II score, a 13.33% rate of co-infections prior to CRE detection, and an uncontrolled source of infection in 33.33% of patients. Furthermore, colistin-resistant *Klebsiella pneumoniae* (CoR-KP) accounted for 30% of the causative CRE isolates in our study. This finding aligns with previous reports, which have documented similarly high 14-day (60.71%) and in-hospital (82.14%) mortality rates in cohorts infected with such difficult-to-treat pathogens [2]. The seemingly high 14-day microbiological eradication rate (88.89%) must be interpreted with caution. Excluding early mortalities introduces survivor bias, as this rate reflects only patients surviving until follow-up cultures.

To the best of our knowledge, this is the first study to explore and propose a potential PK/PD target for HD-TGC therapy in patients with CRE BSIs. Our findings suggest that an AUCss, 0–24 h/MIC ratio of ≥ 9.90 is associated with significantly reduced CRE-attributable mortality and improved microbiological eradication. Given the lack of an established PK/PD target for TGC in CRE BSIs, our findings were compared against cohorts with similar disease severity, such as those with hospital-acquired (HAP) or ventilator-associated pneumonia (VAP). Previous HAP/VAP studies proposed lower PK/PD targets, such as a total AUC/MIC ≥ 4.5 or the free drug area under the concentration-time curve to MIC ratio (*f*AUC/MIC) ≥ 0.9 to predict clinical cure and *f*AUC/MIC ≥ 0.35 to predict microbiological response [26]. Our identified cutoff is substantially higher. This discrepancy is expected, as those earlier studies were based on standard-dose regimens that yield lower overall drug exposures and were conducted in cohorts with lower disease severity. However, our identified threshold of 9.90 aligns closely with findings from a previous HAP/VAP study in which 50% of the patients received HD-TGC and the cohort exhibited high disease severity (mean APACHE II score of 18.97) [14]. The investigators derived a nearly identical total AUC/MIC cutoff of ≥ 10.12 to predict clinical cure. The strong similarity suggests that a total AUC/MIC ratio of approximately 10 is the necessary PK/PD target for achieving clinical success in severe CRE infections treated with high-dose regimens. Furthermore, multivariable analysis revealed that achieving this target significantly reduced 30-day CRE-attributable mortality. Although companion regimens did not significantly impact survival in our model, our study lacks sufficient power to definitively claim they have no effect. Moreover, these findings remain subject to residual confounding from baseline illness severity and the heterogeneous companion antibiotics used. Notably, the concomitant antimicrobials administered, which were selected based on in vitro susceptibility, demonstrated synergistic or additive effects and a lack of antagonism [18, 27, 28]. This stands in contrast to the combination of TGC and ceftazidime-avibactam, where recent in vitro data suggest that TGC may compromise the bactericidal activity of the latter [29]. Nevertheless, this regimen was infrequently used in our cohort. Beyond these strategies, cefiderocol offers broad coverage against all carbapenemase classes [30], and its combination with avibactam may further improve susceptibility in NDM strains [31]. Although cefiderocol-containing regimens have been associated with lower mortality compared to colistin-containing regimens in NDM-producing CRE [10], its broader clinical application for CRE BSIs remains restricted by insufficient efficacy data, limited regional access, and the concerning emergence of resistance [32, 33].

From a microbiological perspective, our in vitro analysis of 30 CRE isolates demonstrated TGC MIC50 and MIC90 values of 0.5 mg/L and 1 mg/L, respectively, with an overall MIC range of 0.25–2.0 mg/L. The clinical interpretation of these MICs is highly dependent on the breakpoints used. According to U.S. FDA criteria, 100% of our isolates would be classified as susceptible. In contrast, when applying the 2025 EUCAST criteria, only 50% of the isolates were susceptible. This marked interpretive discordance highlights the potential pitfalls of relying solely on categorical reporting. Consequently, we strongly advocate for using specific MIC values to guide antimicrobial selection, rather than relying exclusively on breakpoint-defined susceptibility categories, to ensure optimal PK/PD target attainment. Furthermore, our observed MIC values are consistent with those reported in previous studies from 2019 to 2020, which showed an MIC50 of 0.5 mg/L and an MIC90 of 1 or 2 mg/L [6, 11]. This finding suggests that TGC susceptibility patterns among local CRE isolates have remained stable over recent years.

This study integrates in vitro, pharmacokinetic, and clinical data to propose an AUCss, 0–24 h/MIC ratio of ≥ 9.90 as an optimal target to improve the clinical and microbiological outcomes. Our pharmacokinetic analysis revealed a median AUCss, 0–24 h of 14.81 mg·h/L, indicating that the high-dose regimen reliably achieves this therapeutic target for isolates with an MIC is ≤ 1 mg/L. These findings align with prior Monte Carlo simulation of HD-TGC, which showed a PTA ≥ 90% in CR-KP with similar MIC values [34, 35]. However, while HD-TGC combination therapy improved microbiological eradication, it did not confer a significant survival benefit regarding all-cause mortality [36]. Consequently, we recommend that newer beta-lactamase inhibitors remain the first-line regimens as recommended by current guidelines. In settings with limited access to these agents, HD-TGC combination therapy may be considered a viable alternative. Nevertheless, the potential benefits of this aggressive dosing strategy must be weighed against safety concerns. Notably, HD-TGC is associated with dose-dependent adverse events, including hypofibrinogenemia and hepatotoxicity [37, 38]. Therefore, careful monitoring of coagulation parameters and liver function tests is strongly advised when employing HD-TGC in critically ill patients.

This study has several key strengths, including its prospective design, the use of gold-standard methods for in vitro microbiological analysis, and the identification of a potential PK/PD target for HD-TGC in CRE BSIs. However, these findings must be interpreted in the context of several limitations. First, the study was constrained by a small sample size from a single center. Specifically, the low number of outcome events per variable introduces statistical instability, as reflected by the wide confidence intervals for mortality outcomes. Consequently, the odds ratios reported in this study must be interpreted with caution. Furthermore, we also recognize that using ROC and CART analyses with a small cohort carries a high risk of overfitting, and that the resulting high AUC value may be mathematically unstable. Therefore, this exploratory cutoff is preliminary and requires external validation in a larger cohort. Second, although we employed a rich pharmacokinetic sampling strategy for precise AUC estimation, steady-state conditions were not fully achieved in seven patients at the time of sampling. This early sampling may underestimate total AUC exposures, and our limited sample size precluded a sensitivity analysis excluding these patients to evaluate robustness. Third, our PK/PD target relies on total TGC concentrations, and free drug targets were not assessed. Although free TGC fractions may vary in critically ill patients with hypoalbuminemia due to moderate to high, non-linear protein binding [39, 40], quantifying free drug fraction requires resource-intensive techniques. Finally, we did not evaluate the pharmacokinetics and potential synergistic activity of companion drugs, nor did we analyze the genotypes of the causative pathogens.

## Conclusion

Based on our exploratory findings in patients with CRE BSIs receiving HD-TGC combination therapy, we propose a hypothesis-generating AUCss, 0–24 h/MIC target of ≥ 9.90, which was associated with reduced CRE-attributable mortality and improved microbiological eradication. To achieve this target, our data suggest that HD-TGC is most likely to be effective against pathogens with a TGC MIC ≤ 1 mg/L. Further external validation in larger cohorts is required to confirm the clinical utility of this threshold.

## Supplementary Information

Below is the link to the electronic supplementary material.

Supplementary Material 1

## Data Availability

The datasets generated and/or analyzed during the current study are not publicly available due to privacy and ethical restrictions but are available from the corresponding author on reasonable request.

## Abbreviations

AUC: Area under the concentration-time curve
AUCss, 0-24h: Area under the concentration-time curve over the 24-hour dosing interval at steady-state
AUC/MIC: Area under the curve above minimum inhibitory concentration
*f*AUC/MIC: The free drug area under the concentration-time curve to MIC ratio
BSIs: Bloodstream infections
CRE: Carbapenem resistant Enterobacterales
CR-KP: Carbapenem resistant *Klebsiella pneumoniae*
Cmax: Maximum concentration
Ctr: Trough concentration
CART: Classification and regression tree
HD-TGC: High-dose tigecycline
MIC: Minimum inhibitory concentration
MIC50: MIC at the 50th percentile
MIC90: MIC at the 90th percentile
PK/PD: Pharmacokinetics and pharmacodynamics
ROC: Receiver operating characteristic
TGC: Tigecycline
