# Supplementary material for: The pharmacokinetics/pharmacodynamics index of high-dose tigecycline in patients with carbapenem-resistant Enterobacterales bloodstream infection: a prospective study
Source: BMC Infect Dis. 2026 May 20;26:1347. doi: 10.1186/s12879-026-13601-2 (PMC13371137; doi:10.1186/s12879-026-13601-2)
Supplement: Supplementary file 1 — Supplementary Material 1 [file 12879_2026_13601_MOESM1_ESM.docx]

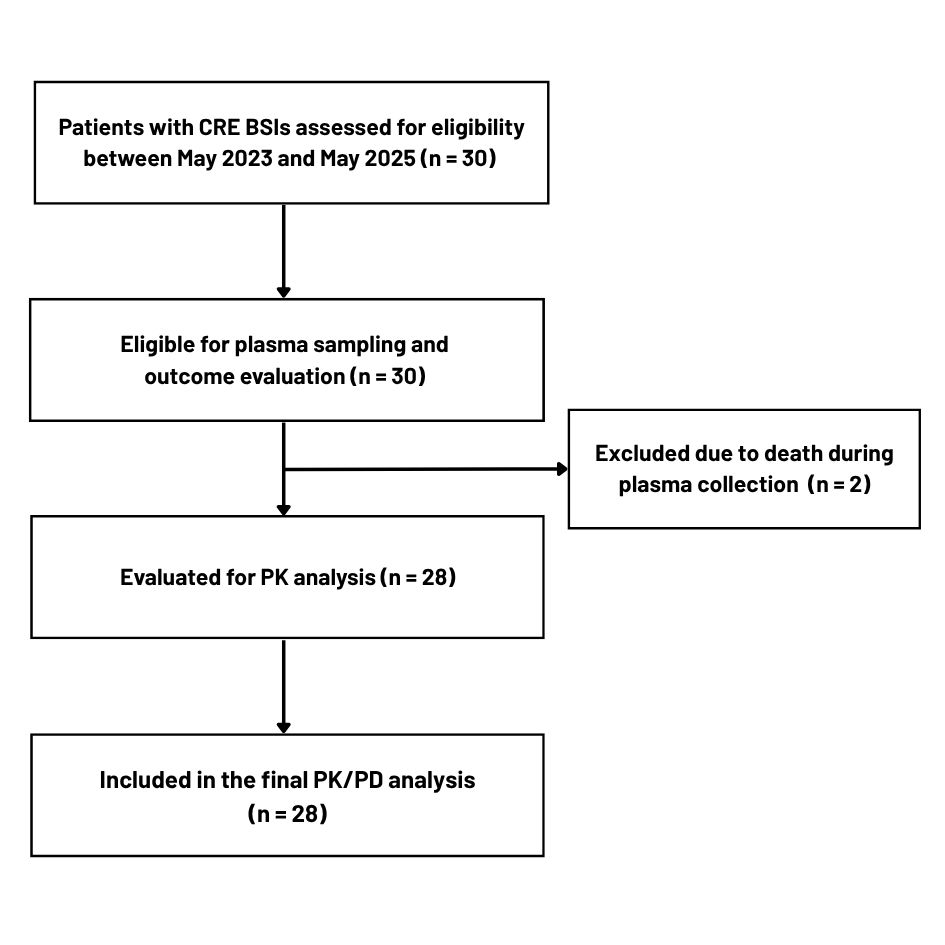


Supplementary Figure S1: Flowchart of patient enrollment

Supplementary Table 1: Pharmacokinetic parameters of high-dose tigecycline in 28 patients

| **Parameters** | **Mean** | **Median** | **Standard deviation** | **Interquartile range** |
| --- | --- | --- | --- | --- |
| Cmax (mg/L) | 0.83 | 0.60 | 0.57 | 0.42-1.24 |
| Ctr (mg/L) | 0.23 | 0.17 | 0.16 | 0.13-0.29 |
| Vd (L) | 311.16 | 250.73 | 221.20 | 147.34-412.56 |
| CL (L/h) | 15.90 | 13.51 | 9.89 | 8.51-21.10 |
| Half-life (h) | 17.45 | 11.96 | 14.67 | 8.17-22.66 |
| AUCss, 0-24h (h⋅mg/L) | 19.47 | 14.81 | 16.56 | 9.48-23.50 |

Abbreviations: Cmax, maximum concentration; Ctr, trough concentration; Vd, volume of distribution; CL, clearance; AUCss, 0-24h, area under the concentration-time curve over the 24-hour dosing interval at steady-state.

Supplementary Table 2: Clinical and microbiological outcomes stratified by tigecycline minimum inhibitory concentration (MIC)

| Clinical outcomes  MIC of TGC | Number of patients | 0.25 mg/L  (N=8) | 0.50 mg/L  (N=7) | 1.0 mg/L  (N=12) | 2.0 mg/L  (N=3) |
| --- | --- | --- | --- | --- | --- |
| 7-day all-cause mortality | 5/30 | 2/8  (25.00%) | - | 3/12  (25.00%) | - |
| 14-day all-cause mortality | 15/30 | 4/8  (50.00%) | 3/7  (42.86%) | 7/12  (58.33%) | 1/3  (33.33%) |
| 30-day all-cause mortality | 24/30 | 5/8  (62.50%) | 6/7  (85.71%) | 10/12  (83.33%) | 3/3  (100%) |
| 30-day CRE-attributable mortality | 10/30 | 1/8  (12.50%) | 3/7  (42.86%) | 4/12  (33.33%) | 2/3 (66.67%) |
| In-hospital CRE-attributable mortality | 12/30 | 2/8  (25.00%) | 3/7  (42.86%) | 5/12  (41.67%) | 2/3 (66.67%) |
| 7-day clinical cure | 21/30 | 5/8  (62.50%) | 7/7  (100%) | 6/12  (50.00%) | 3/3  (100%) |
| 14-day clinical cure | 11/30 | 4/8  (50.00%) | 3/7  (42.86%) | 3/12  (25.00%) | 1/3  (33.33%) |
| 7-day microbiological cure | 20/28 | 6/8  (75.00%) | 6/7  (85.71%) | 5/10  (50.00%) | 3/3  (100%) |
| 14-day microbiological cure | 16/18 | 5/5  (100%) | 4/4  (100%) | 6/7  (85.71%) | 1/2  (50.00%) |

Supplementary Table 3: Univariable and multivariable logistic regression analyses of factors associated with 30-day CRE-attributable mortality

| **Factors** | **Death**  **N=10** | **Alive**  **N=20** | **Univariate analysis** | | | **Multivariate analysis** | | |
| --- | --- | --- | --- | --- | --- | --- | --- | --- |
|  |  |  | **OR** | **p-value** | **95%CI** | **OR** | **p-value** | **95%CI** |
| Male | 8/10 (80.00) | 17/20 (85.0) | 0.71 | 0.73 | 0.098-5.096 |  |  |  |
| Age | 83 (64.00-87.00) | 72 (58.00-80.25) | 1.05 | 0.12 | 0.987-1.124 |  |  |  |
| CCI | 4.50 (3.75-6.50) | 5.0 (4.00-6.75) | 1.02 | 0.91 | 0.730-1.423 |  |  |  |
| SOFA | 10.00 (7.50 -12.50) | 8.00 (6.00-11.00) | 1.14 | 0.26 | 0.910-1.415 |  |  |  |
| APACHE II | 31.00 (25.75-34.25) | 24.50 (18.75-29.75) | **1.21** | **0.03** | **1.016-1.440** | 1.24 | 0.07 | 0.987-1.560 |
| Septic shock | 7/10 (70.00) | 10/20 (50.0) | 2.33 | 0.30 | 0.466-11.693 |  |  |  |
| Mechanical ventilation | 7/10 (70.00) | 14/20 (70.0) | 1.00 | 1.00 | 0.191-5.241 |  |  |  |
| MIC of TGC ≥1 mg/L | 6/10 (60.00) | 9/20 (45.0) | 1.83 | 0.44 | 0.392-8.566 |  |  |  |
| Source control | 6/10 (60.00) | 14/20 (70.00) | 0.64 | 0.59 | 0.132-3.140 |  |  |  |
| AUC_ss, 0-24h_/MIC ≥ 9.90 | 4/8 (50.00) | 19/20 (95.00) | **0.05** | **0.02** | **0.005-0.605** | **0.04** | **0.03** | **0.002-0.780** |
| TGC+COL (N=14) vs non TGC+COL (N=16) | 5/10 (50.00) | 9/20 (45.00) | 1.22 | 0.80 | 0.267-5.592 |  |  |  |
| TGC+AMK (N=8) vs  non TGC+AMK (N=22) | 4/10 (40.00) | 4/20 (20.00) | 2.67 | 0.25 | 0.500-14.217 |  |  |  |

Note: Data are presented as median (interquartile range [IQR]) for continuous variables and number (percentage) for categorical variables.

Abbreviations: CCI, Charlson Comorbidity Index; APACHE II, Acute Physiology and Chronic Health Evaluation II; SOFA, Sequential Organ Failure Assessment; TGC+COL, tigecycline plus colistin; TGC+AMK, tigecycline plus amikacin; OR, odds ratio; 95% CI, 95% confidence interval.
